# Is the Sokal or EUTOS long-term survival (ELTS) score a better predictor of responses and outcomes in persons with chronic myeloid leukemia receiving tyrosine-kinase inhibitors?

**DOI:** 10.1038/s41375-021-01387-y

**Published:** 2021-08-19

**Authors:** Xiao-Shuai Zhang, Robert Peter Gale, Xiao-Jun Huang, Qian Jiang

**Affiliations:** 1 Peking University People’s Hospital, Peking University Institute of Hematology, National Clinical Research Center for Hematologic Disease, Beijing Key Laboratory of Hematopoietic Stem Cell Transplantation, Beijing, PR China; 2 Centre for Haematology Research, Department of Immunology and Inflammation, Imperial College London, London, UK; 3 Collaborative Innovation Center of Hematology, Soochow University, Suzhou, PR China; 4 Peking-Tsinghua Center for Life Sciences, Academy for Advanced Interdisciplinary Studies, Peking University, Beijing, China

**Keywords:** Chronic myeloid leukaemia, Targeted therapies

## Abstract

Data from 1661 consecutive subjects with chronic-phase chronic myeloid leukemia (CML) receiving initial imatinib (*n* = 1379) or a 2^nd^-generation tyrosine-kinase inhibitor (2G-TKI; *n* = 282) were interrogated to determine whether the Sokal or European Treatment and Outcome Study for CML (EUTOS) long-term survival (ELTS) scores were more accurate responses and outcome predictors. Both scores predicted probabilities of achieving complete cytogenetic response (CCyR), major molecular response (MMR), failure- and progression-free survivals (FFS, PFS), and survival in all subjects and those receiving imatinib therapy. However, the ELTS score was a better predictor of MR^4^, MR^4.5^, and CML-related survival than the Sokal score. In subjects receiving 2G-TKI therapy, only the ELTS score accurately predicted probabilities of CCyR, MMR, MR^4^, FFS, and PFS. In the propensity score matching, subjects classified as intermediate risk by the ELTS score receiving a 2G-TKI had better responses (*p* < 0.001~0.061), FFS (*p* = 0.002), and PFS (*p* *=* 0.03) but not survival. Our data suggest better overall prediction accuracy for the ELTS score compared with the Sokal score in CML patients, especially those receiving 2G-TKIs. People identified as intermediate risk by the ELTS score may benefit more from initial 2G-TKI therapy in achieving surrogate endpoints but not survival, especially when a briefer interval to stopping TKI therapy is the therapy objective.

## Introduction

Several risk scores have been developed to predict responses and/or outcomes of persons with chronic-phase chronic myeloid leukemia (CML). However, predictive scores are only accurate in the context of the therapy given (as opposed to prognostic scores). For example, the Sokal and Hasford scores were developed in persons receiving chemotherapy and/or interferon [1, 2]. The accuracy of these scores in persons receiving TKI-therapy is controversial [3–6]. In contrast, the European Treatment and Outcome Study for CML (EUTOS) and EUTOS long-term survival (ELTS) scores was developed in persons receiving predominately imatinib [7, 8]. The Sokal and ELTS scores are the most commonly used today in persons receiving TKI therapy. Several studies reported that the ELTS score is more accurate in identifying high-risk populations and better ability to predict CML-related deaths and survival in persons receiving imatinib or a 2^nd^-generation TKI (2G-TKI) [9–16]. The ELTS score is also a more accurate predictor of the probability of achieving a complete cytogenetic response (CCyR) and major molecular response (MMR) [11, 16]. Consequently, the ELTS score is preferred in the 2020 European LeukemiaNet (ELN) recommendations [17].

Few studies critically compared the Sokal and ELTS scores as predictors of cytogenetic and molecular responses and other outcomes such as failure- and progression-free survivals (FFS and PFS), especially in persons receiving 2G-TKIs recommended by some for persons with intermediate- or high-risk CML [18]. We compared prediction accuracies of the Sokal and ELTS scores on responses and outcomes in 1661 consecutive subjects with chronic-phase CML receiving imatinib or a 2G-TKI. We found better overall prediction accuracy for the ELTS score. People identified as intermediate risk in the ELTS score may benefit more from 2G-TKI therapy compared with imatinib in achieving surrogate endpoints but not CML-related survival or survival, especially when a briefer interval to stopping TKI therapy is the therapy objective.

## Subjects and methods

### Subjects

We interrogated data from 1661 consecutive newly diagnosed subjects with chronic-phase CML ≥ 18 years receiving imatinib, dasatinib, or nilotinib therapy at Peking University People’s Hospital from January 2006 to March 2021. Data of covariates determined at diagnosis included sex, age, comorbidities, hemoglobin concentration, WBC and platelet counts, cytogenetic analyses, and initial TKI therapy. Sokal and ELTS scores at diagnosis were calculated as described [2, 8]. Therapy responses and outcomes were extracted from medical records. Physicians and patients jointly choose the initial TKI given based on which TKIs were available, anticipated safety and efficacy, and economics. The initial imatinib dose was 400 mg daily; nilotinib, 300 mg twice daily; dasatinib, 100 mg daily. Dose and/or type of TKI were adjusted during therapy based on responses, adverse events, and operative ELN recommendations [17, 19–21]. The study was approved by the Ethics Committee of Peking University People’s Hospital compliant with the Helsinki Declaration. Subjects gave written informed consent.

### Diagnosis, monitoring, responses, and outcomes

Diagnosis, monitoring, and therapy responses conformed operative ELN recommendations [17, 19–21]. Bone marrow cytogenetic analyses used G-banding. *BCR*::*ABL1* transcript levels in blood were assessed by quantitative real time polymerase chain (qRT-PCR) with *ABL1* as control and converted to international scales (*BCR::ABL1*^*IS*^) using our laboratory-specific conversion factor of 0.65 (Institute of Medical and Veterinary Science International Reference Laboratory, Adelaide, Australia) [22].

Response assessment was performed on the *intention-to-treat* population. Haematologic response was monitored every 1–2 weeks, until a complete hematologic response (CHR) and every 3–6 months thereafter. The cytogenetic response was assessed at baseline and then every 3–6 months, until a CCyR was achieved and repeated at therapy failure. High-risk additional cytogenetic abnormalities (ACAs) were defined according to 2020 ELN criteria [17]. Molecular monitoring was done at baseline and every 3 months, until major molecular response (MMR) and every 3–6 months thereafter. Screening for *ABL1* mutation was done in subjects with a *suboptimal* or *warning* response according to operative ELN criteria [17, 19–21].

Responses and outcomes were defined as follows: (1) CCyR, no Ph [1]-positive cells in ≥ 20 bone marrow cell metaphases; (2) MMR, *BCR*::*ABL1*^*IS*^ ≤ 0.1%; (3) molecular response 4 (MR^4^), *BCR::ABL1*^*IS*^ ≤ 0.01%; (4) molecular response 4.5 (MR^4.5^), *BCR*::*ABL1*^*IS*^ ≤ 0.0032%; (5) therapy failure, according to the 2020 ELN criteria, including failing to meet the ELN time-dependent *BCR::ABL1* transcript levels, development of *ABL1* mutations and/or high-risk additional cytogenetic abnormalities, or progression to accelerated and/or blast phases [17].

### Statistical analyses

Descriptive statistics were used to summarize covariates. Categorical variables are reported as percentages and counts and continuous variables as medians and ranges. Pearson chi-squared test (for categorical variables) and Mann–Whitney U test (for continuous variables) were used to compare the imatinib and 2G-TKI cohorts. Cumulative incidences of CCyR, MMR, MR^4^, and MR^4.5^ were calculated using the Fine-Gray test that considered competing events such as death, transplant, loss to follow-up, and/or withdrawal of consent. Failure- and progression-free survivals (FFS, PFS), CML-related survival, and survival were calculated using the Kaplan-Meier estimator and log-rank tests.

Potential predictive covariates for diverse responses and outcomes were tested in univariable analyses and those with *p* < 0.2 were included in multivariable analyses using a backward-elimination process to fit a Cox regression model. Cox regression models were built to identify independent covariates associated with responses and outcomes reported as hazard ratios (HRs) with 95% confidence intervals (CIs).

FFS was calculated from TKI-therapy start to therapy failure or censored at the last follow-up. PFS was calculated as TKI-therapy start to progression, death at any time, or censored at the last follow-up. CML-related survival was calculated from TKI-therapy start to death from CML progression or censored at the last follow-up. Survival was calculated as TKI therapy to death from any cause or censored at the last follow-up.

Propensity-score matching was used to explore whether the Sokal or ELTS score was a better predictor of responses and outcomes to imatinib or 2G-TKI as 1^st^ therapy, including all covariates tested in the univariable analyses. Covariate balance was evaluated using the standardized absolute mean difference (SAMD). SAMD < 0.02 was considered adequate balance. A two-sided test with *p* < 0.05 was considered statistically significant. SPSS 22.0 (SPSS, Chicago, IL, USA), R version 4.0.2 (R Core Team, Vienna, Austria), and GraphPad Prism 8 (GraphPad Software Inc., La Jolla, CA, USA) were used for analyses and graphing.

## Results

### Subjects

**Fig. 1:**
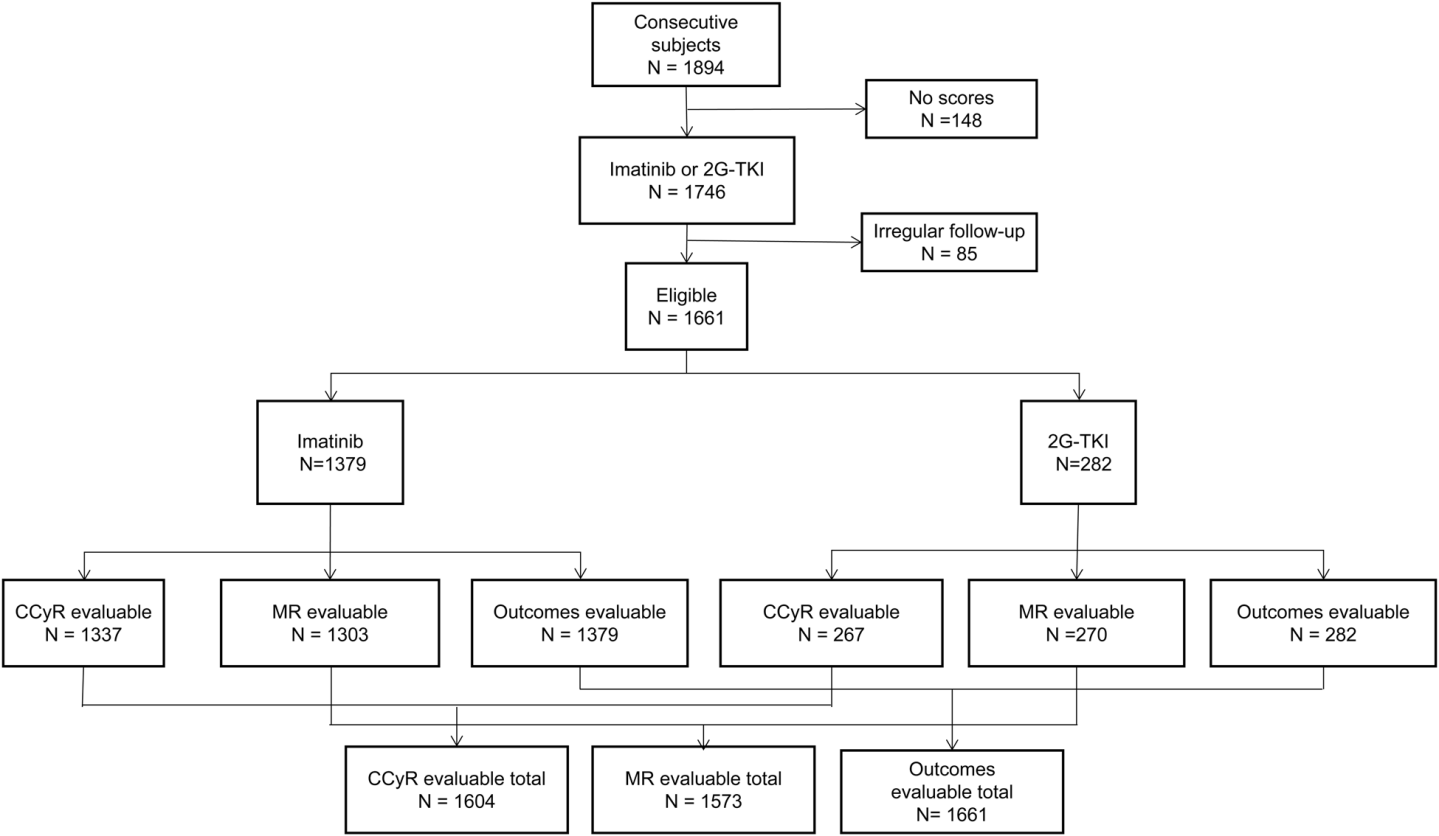
Study flow diagram.

In total, 1894 consecutive subjects were interrogated. In total, 233 were excluded because there were no Sokal and ELTS scores (*n* = 148) and irregular follow-up (*n* = 85). The 1661 remaining subjects initially received imatinib (*n* = 1379; 83%), nilotinib (*n* = 206; 12%), or dasatinib (*n* = 76; 5%; Fig. 1); 1021 subjects (62%) were male. Median age was 40 years (interquartile range [IQR], 29–51 years). In total, 605 (36%) had ≥1 comorbidity and 18, ≥1 high-risk ACAs. In total, 755 (46%), 522 (31%), and 384 (23%) were classified as low-, intermediate-, or high risk using the Sokal score. Similar assignments using the ELTS score were 1098 (66%), 414 (25%), and 149 (9%). In total, 1633 subjects (98%) had *e13a2* and/or *e14a2 BCR*::*ABL1* and 28 subjects (2%), other transcripts.

Subjects initially receiving a 2G-TKI therapy were younger (*p* < 0.001), had the higher WBC counts (*p* = 0.001), lower hemoglobin concentration (*p* < 0.001), higher percentages of blood blasts (*p* = 0.001), and basophils (*p* = 0.003), and were more likely high-risk using Sokal and ELTS scores (*p* < 0.001; Table 1). Median follow-up is 60 months (IQR, 33–85 months) in the imatinib cohort and 46 months (IQR, 19–68 months) in the 2G-TKI cohort (*p* < 0.001) because of the later availability of 2G-TKIs. In total, 1074 subjects (78%) in the imatinib cohort and 247 in the 2G-TKI cohort remained on their 1st TKI (79% vs. 88%; *p* = 0.18). In total, 298 subjects (22%) receiving initial imatinib switched to nilotinib (*n* = 224), dasatinib (*n* = 66), or, olverembatinib (*n* = 8) as their 2nd (*n* = 257) or 3rd (*n* = 33), TKI because of therapy failure (*n* = 242), adverse events (*n* = 33) or by choice (*n* = 23). 33 subjects (12%) receiving initial 2G-TKI therapy switched to imatinib (*n* = 27) or olverembatinib (*n* = 6) as 2nd (*n* = 31) or 3rd (*n* = 2) TKI because of cost (*n* = 17), adverse events (*n* = 12), or therapy failure (*n* = 4). In total, 9 subjects receiving initial imatinib (*n* = 7) or 2G-TKI (*n* = 2) discontinued TKI therapy after achieving ≥ MR^4^. In total, 19 subjects were lost to follow-up totally.

**Table 1:** Subject covariates.

Variable	All (*N* = 1661)	Imatinib (*n* = 1379)	2G-TKIs (*n* = 282)	*p-*value
Age, years	40 (18, 83)	41 (18, 83)	35 (18, 73)	<0.001
Sex				0.721
Male	1021 (61.5%)	845 (61.3%)	176 (62.4%)	
Sokal risk				<0.001
Low	755 (45.5%)	651 (47.2%)	104 (36.9%)	
Intermediate	522 (31.4%)	436 (31.6%)	86 (30.5%)	
High	384 (23.1%)	292 (21.2%)	92 (32.6%)	
ELTS risk				<0.001
Low	1098 (66.1%)	937 (67.9%)	161 (57.1%)	
Intermediate	414 (24.9%)	334 (24.2%)	80 (28.4%)	
High	149 (9.0%)	108 (7.8%)	41 (14.5%)	
WBC, ×10E + 9/L	120 (3, 786)	112 (5, 786)	155 (5, 755)	0.001
Haemoglobin, g/L	115 (28, 183)	116 (28, 183)	109 (57, 167)	<0.001
Platelets, ×10E + 9/L	406 (28, 3707)	401 (36, 3707)	439 (28, 2887)	0.130
Blood blasts, %	1 (0, 14)	1 (0, 14)	1 (0, 13)	0.001
Blood basophils, %	4 (0, 19)	4 (0, 19)	5 (0, 19)	0.003
Ph^+^ ACAs				0.257
Yes	50 (3.0%)	36 (2.6%)	14 (5.0%)	
No	1042 (84.4%)	1163 (84.3%)	239 (84.8%)	
Unknown	209 (12.6%)	180 (13.1%)	29 (10.3%)	
≥ 1 Co-morbidity(ies)	605 (36.4%)	510 (37.0%)	95 (33.7%)	0.295
Follow-up, months	58 (3, 193)	60 (3, 193)	46 (3, 160)	<0.001

The data are presented as the number (%) or median (range), except where otherwise noted.

2G-TKI second-generation tyrosine kinase inhibitor, Ph^+^ ACA additional chromosomal aberrations in Philadelphia-positive cells, PLT platelet, TKI tyrosine kinase inhibitor, WBC white blood cell.

### All subjects

**Fig. 2:**
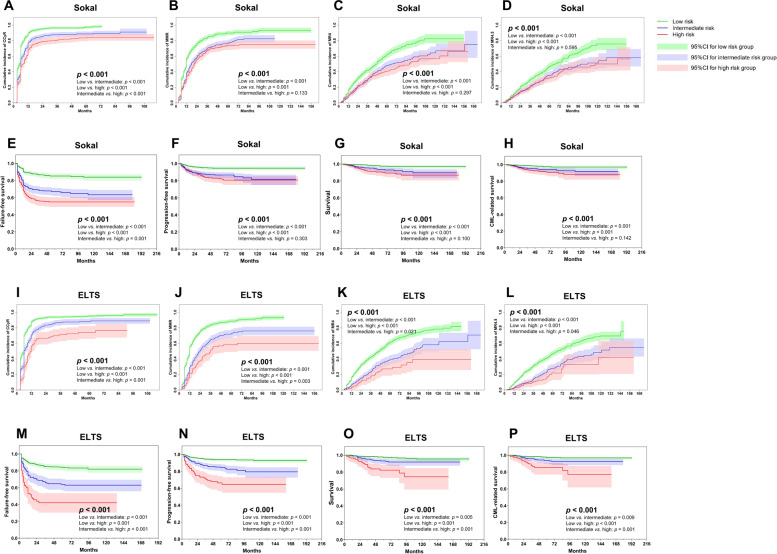
Therapy responses and outcomes of all subjects. **A**–**D** CCyR, MMR, MR^4^, and MR^4.5^ by Sokal score, **E**–**H** FFS, PFS, survival, and CML-related survival by Sokal score, **I–L** CCyR, MMR, MR^4^, and MR^4.5^ by ELTS score, **M**–**P** FFS, PFS, survival, and CML-related survival by ELTS score.

In total, 1614 subjects (97%) achieved a CHR. In total, 1604 subjects (97%) were studied for CCyR. In total, 1573 with common *BCR*::*ABL1* transcripts were studied for MMR, MR^4^, and MR^4.5^. In total, 1464 (91% [95% Confidence Interval (CI), 87, 95%]), 1208 (77% [73, 81%]), 1070 (68% [63, 74%]), and 1002 (64% [58, 70%]) achieved a CCyR, MMR, MR^4^, and MR^4.5^. In total, 419 subjects (25% [22, 28%]) had therapy failure, 156 (9% [7, 11%]) transformed to accelerated (*n* = 88, 5% [4, 6%]) or blast phases (*n* = 68, 4% [3, 5%]), and 75 subjects (5% [3, 6%]) died of transformation to accelerated or blast phases (*n* = 68, 4% [3, 5%]) or other causes (*n* = 7, < 1%). In all, 7-year cumulative incidences of CCyR, MMR, MR^4^, and MR^4.5^ were 92% (87, 96%), 83% (77, 91%), 65% (61, 69%), and 54% (39, 68%). About 7-year probabilities of FFS, PFS, CML-related survival, and survival were 72% (70, 74%), 89% (87, 92%), 94% (92, 95%), and 94% (92, 95%; Fig. 2).

Subject covariates indicated above were analyzed to explore whether the Sokal or ELTS score was a better predictor of CCyR, MMR, MR^4^, MR^4.5^, FFS, PFS, survival, and CML-related survival. There were no interactions between these covariates (VIFs = 1.0–1.7). In multivariable analyses, both scores were significantly associated with the probabilities of CCyR (Sokal, *p* < 0.001; ELTS, *p* = 0.001), MMR (Sokal, *p* = 0.003; ELTS, *p* < 0.001), FFS (Sokal, *p* < 0.001; ELTS: *p* < 0.001), PFS (Sokal, *p* = 0.008; ELTS, *p* < 0.001), and survival (Sokal, *p* = 0.063; ELTS, *p* < 0.001). However, only the ELTS score was significantly associated with the cumulative incidence of MR^4^ (intermediate vs. low, hazard ratio [HR] =0.8 [0.6, 1.0], *p* = 0.028; high vs. low, HR = 0.6 [0.4, 0.9], *p* = 0.013), MR^4.5^ (intermediate vs. low, HR = 0.8 [0.6, 1.0], *p* = 0.041; high vs. low, HR = 0.6 [0.4, 0.9], *p* = 0.030), and CML-related survival (high vs. low, HR = 4.3 [2.3, 8.1], *p* < 0.001). Male sex, lower hemoglobin concentration, higher WBC counts, and initial imatinib therapy were significantly associated with lower probabilities of molecular responses and/or inferior outcomes (Table 2).

**Table 2:** Multi-variable analyses.

Variable	CCyR		MMR		MR^4^		MR^4.5^		FFS		PFS		Survival		CML-related survival	
	HR (95%CI)	*p* value	HR (95%CI)	*p* value	HR (95%CI)	*p* value	HR (95%CI)	*p* value	HR (95%CI)	*p* value	HR (95%CI)	*p* value	HR (95%CI)	*p* value	HR (95%CI)	*p* value
All subjects																
Sokal risk		<0.001		0.003						<0.001		0.008		0.063		
Low (ref.)																
Intermediate	0.8 (0.7, 0.9)	0.004	0.8 (0.7, 0.9)	0.002					1.7 (1.2, 2.3)	0.001	2.2 (1.3, 3.8)	0.002	2.3 (1.1, 4.6)	0.026		
High	0.7 (0.6, 0.8)	<0.001	0.8 (0.7, 0.9)	0.012					2.0 (1.4, 2.8)	<0.001	1.6 (0.9, 2.9)	0.091	1.8 (0.8, 3.9)	0.143		
ELTS risk		0.001		<0.001		0.010		0.022		<0.001		<0.001		<0.001		<0.001
Low (ref.)																
Intermediate	0.9 (0.7, 1.0)	0.080	0.8 (0.6, 0.9)	0.003	0.8 (0.6, 1.0)	0.028	0.8 (0.6, 1.0)	0.041	1.5 (1.1, 2.0)	0.004	1.7 (1.0, 2.7)	0.035	1.8 (1.0, 3.2)	0.063	1.4 (0.8, 2.7)	0.227
High	0.6 (0.5, 0.8)	<0.001	0.6 (0.4, 0.8)	<0.001	0.6 (0.4, 0.9)	0.013	0.6 (0.4, 0.9)	0.030	2.5 (1.8, 3.5)	<0.001	4.0 (2.3, 7.0)	<0.001	5.0 (2.7, 9.1)	<0.001	4.3 (2.3, 8.1)	<0.001
Female (ref. male)			1.4 (1.2, 1.6)	<0.001	1.2 (1.0, 1.4)	0.028	1.5 (1.2, 1.8)	<0.001								
WBC, ×10E + 9/L (Continuous)	0.9 (0.9, 1.0)	0.069	0.9 (0.8, 0.9)	<0.001	0.8 (0.7, 0.9)	<0.001	0.7 (0.6, 0.8)	<0.001			0.9 (0.7, 1.0)	0.053				
Haemoglobin, g/L (Continuous)	1.1 (1.0, 1.1)	<0.001	1.1 (1.1, 1.1)	<0.001	1.0 (1.0, 1.0)	<0.001	1.1 (1.0, 1.1)	0.003	0.9 (0.8, 0.9)	<0.001	0.8 (0.7, 0.9)	<0.001	0.9 (0.8, 1.0)	0.009	0.8 (0.8, 0.9)	0.004
2G-TKI (ref. Imatinib)	1.6 (1.4, 1.9)	<0.001	1.4 (1.2, 1.7)	<0.001	1.4 (1.1, 1.7)	0.002	1.5 (1.2, 1.9)	0.002								
Imatinib cohort																
Sokal risk		<0.001		0.073						<0.001		0.004		0.058		
Low (ref.)																
Intermediate	0.8 (0.7, 0.9)	0.007	0.9 (0.8, 1.0)	0.125					2.2 (1.6, 3.1)	<0.001	2.3 (1.4, 3.8)	0.001	2.8 (1.2, 6.4)	0.014		
High	0.7 (0.6, 0.8)	<0.001	0.8 (0.7, 1.0)	0.030					2.6 (1.8, 3.7)	<0.001	1.5 (0.9, 2.7)	0.156	2.5 (1.0, 6.0)	0.069		
ELTS risk		0.001		<0.001		0.001		0.017		<0.001		<0.001		<0.001		<0.001
Low (ref.)																
Intermediate	0.9 (0.7, 1.0)	0.071	0.7 (0.6, 0.9)	<0.001	0.7 (0.5, 0.9)	0.001	0.7 (0.5, 1.0)	0.032	1.3 (1.0, 1.8)	0.003	1.7 (1.0, 2.7)	0.035	1.8 (1.0, 3.2)	0.063	1.7 (0.8, 3.4)	0.137
High	0.6 (0.5, 0.8)	<0.001	0.8 (0.5, 0.9)	0.011	0.6 (0.4, 0.9)	0.027	0.5 (0.3, 0.9)	0.029	2.2 (1.5, 3.2)	<0.001	3.9 (2.2, 6.7)	<0.001	5.0 (2.7, 9.1)	<0.001	5.1 (2.5, 10.5)	<0.001
Female (ref. male)			1.3 (1.1, 1.5)	<0.001	1.2 (1.1, 1.4)	0.023	1.4 (1.2, 1.8)	<0.001	0.8 (0.6, 0.9)	0.032						
WBC, ×10E + 9/L (Continuous)	0.9 (0.9, 1.0)	0.002	0.9 (0.8, 0.9)	<0.001	0.8 (0.7, 0.9)	<0.001	0.6 (0.5, 0.7)	<0.001								
Haemoglobin, g/L (Continuous)	1.1 (1.0, 1.2)	<0.001	1.1 (1.1, 1.2)	<0.001	1.1 (1.0, 1.2)	0.001	1.1 (1.0, 1.1)	0.003	0.8 (0.8, 0.9)	<0.001	0.8 (0.7, 0.9)	<0.001	0.8 (0.7, 0.9)	<0.001	0.8 (0.7, 0.9)	0.003
2G-TKI cohort																
ELTS risk		<0.001		0.007		0.039				<0.001		0.003				
Low (ref.)																
Intermediate	0.7 (0.5, 1.0)	0.033	1.0 (0.7, 1.3)	0.623	0.9 (0.6, 1.4)	0.668			1.1 (0.6, 2.0)	0.799	1.2 (0.5, 3.2)	0.703				
High	0.4 (0.2, 0.6)	<0.001	0.4 (0.3, 0.7)	0.002	0.4 (0.2, 0.9)	0.024			3.5 (1.9, 6.2)	<0.001	4.1 (1.7, 9.9)	0.002				
Female (ref. male)			1.5 (1.1, 2.0)	0.008	1.5 (1.1, 1.9)	0.009	1.6 (1.1, 2.3)	0.020								
WBC, ×10E + 9/L (Continuous)					0.9 (0.9, 1.0)	<0.001	0.7 (0.6, 0.8)	<0.001								
Haemoglobin, g/L (Continuous)	1.1 (1.0, 1.2)	0.055	1.1 (1.0, 1.2)	0.005												

*CCyR* complete cytogenetic response, CI confidence interval, FFS failure-free survival, HR hazard ratio, MMR major molecular response, MR^4.5^ molecular response 4.5, PFS progression-free survival.

### Imatinib cohort

**Fig. 3:**
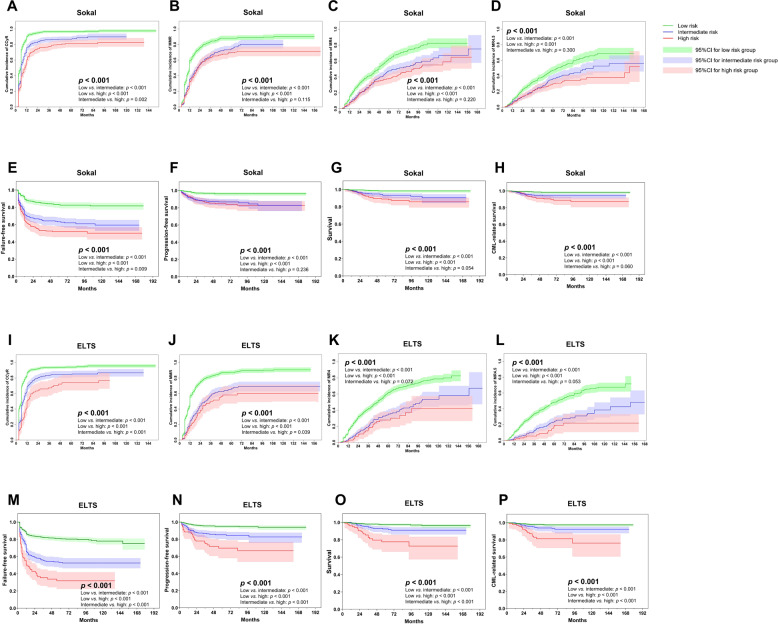
Therapy responses and outcomes of subjects receiving initial imatinib. **A**–**D** CCyR, MMR, MR^4^, and MR^4.5^ by Sokal score, **E-H** FFS, PFS, survival, and CML-related survival by Sokal score, **I**–**L** CCyR, MMR, MR^4^, and MR^4.5^ by ELTS score, **M**–**P** FFS, PFS, survival, and CML-related survival by ELTS score.

In total, 1337 of 1379 subjects (97%) receiving initial imatinib were studied CCyR.1303 with common *BCR*::*ABL1* transcripts were studied for MMR, MR^4^, and MR^4.5^. 1221 (91% [88, 94%]), 1003 (77% [73, 80%]), 928 (71% [66, 74%]), and 889 (68% [63, 72%]) achieved a CCyR, MMR, MR^4^, and MR^4.5^. In total 349 (26% [23, 28%]) had therapy failure, 127 (9% [8, 11%]) transformed to accelerated (*n* = 67, 5% [4, 6%]) or blast phases (*n* = 60, 4% [3, 5%]), and 62 subjects (5% [3, 6%]) died of CML transformation to accelerated or blast phases (*n* = 56, 4% [3, 5%]) or other causes (*n* = 6, < 1%). About 7-year cumulative incidences of CCyR, MMR, MR^4^, and MR^4.5^ were 90% (85, 96%), 78% (74, 83%), 54% (45, 66%), and 43% (39, 54%). About 7-year probabilities of FFS, PFS, CML-related survival and survival were 70% (65, 77%), 89% (83, 94%), 95% (92, 97%), and 94% (91, 97%; Fig. 3).

In multivariable analyses, both scores were significantly associated with the probabilities of CCyR (Sokal, *p* < 0.001; ELTS, *p* = 0.001), MMR (Sokal, *p* = 0.073; ELTS, *p* < 0.001), FFS (Sokal, *p* < 0.001; ELTS: *p* < 0.001), PFS (Sokal, *p* = 0.004; ELTS, *p* < 0.001), and survival (Sokal, *p* = 0.058; ELTS, *p* < 0.001). However, only the ELTS score was significantly associated with the cumulative incidence of MR^4^ (intermediate vs. low, HR = 0.7 [0.5, 0.9], *p* = 0.001; high vs. low, HR = 0.6 [0.4, 0.9], *p* = 0.027), MR^4.5^ (intermediate vs. low, HR = 0.7 [0.5, 1.0], *p* = 0.032; high vs. low, HR = 0.5 [0.3, 0.9], *p* = 0.029), and CML-related survival (high vs. low, HR = 5.1 [2.5, 10.5], *p* < 0.001). Male sex, lower hemoglobin concentration, and higher WBC counts were significantly associated with lower probabilities of molecular responses and/or inferior outcomes (Table 2).

### 2G-TKI cohort

**Fig. 4:**
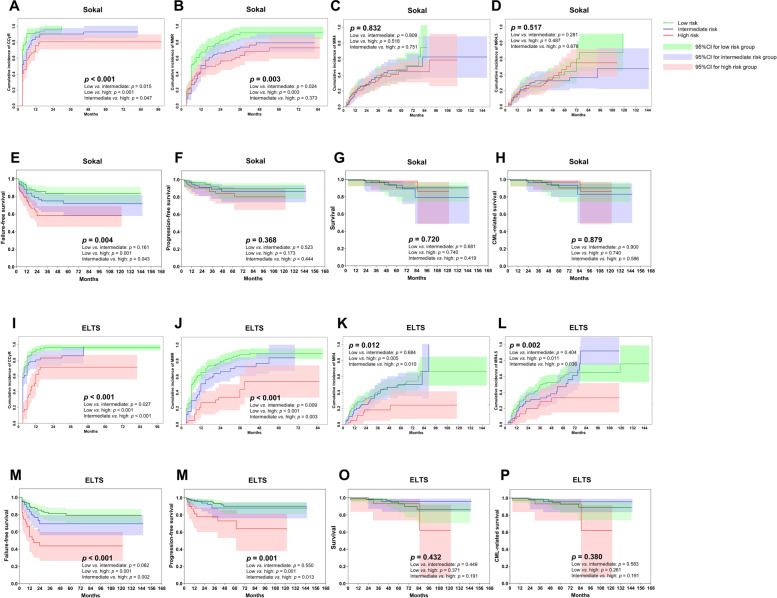
Therapy responses and outcomes of subjects receiving initial 2G-TKIs. **A**–**D** CCyR, MMR, MR^4^, and MR^4.5^ by Sokal score, **E**–**H** FFS, PFS, survival and CML-related survival by Sokal score, **I**–**L** CCyR, MMR, MR^4^, and MR^4.5^ by ELTS score, **M**–**P** FFS, PFS, survival, and CML-related survival by ELTS score.

In total, 267 of 282 subjects (95%) receiving initial 2G-TKI were studied for CCyR. In total, 270 with common *BCR*::*ABL1* transcripts were studied for MMR, MR^4^, and MR^4.5^. In total, 243 (91% [85, 97%]), 205 (76% [69, 82%]), 142 (53% [46, 60%]), and 113 (40% [34, 46%]) achieved CCyR, MMR, MR^4^, and MR^4.5^. In total, 70 (25% [20, 30%]) had therapy failure, 29 (10% [7, 14%]) transformed to accelerated (*n* = 21, 7% [6, 8%]) or blast phases (*n* = 8; 3% [2, 4%]), and 13 (5% [2, 7%]) died of CML progression (*n* = 12, 4% [2, 7%]) or other causes (*n* = 1, <1%). About 5-year cumulative incidences of CCyR, MMR, MR^4^, and MR^4.5^ were 91% (86, 97%), 78% (67, 84%), 58% (49, 67%), and 42% (30, 59%). About 5-year probabilities of FFS, PFS, CML-related survival, and survival were 71% (57, 84%), 87% (72, 96%), 92% (71, 98%), and 91% (71, 99%; Fig. 4).

In multivariable analyses, only the ELTS score predicted probabilities of CCyR (intermediate vs. low, HR = 0.7 [0.5, 1.0]; *p* = 0.033; high vs. low, HR = 0.4 [0.2, 0.6], *p* < 0.001), MMR (high vs. low, HR = 0.4 [0.3, 0.7], *p* = 0.002), and MR^4^ (high vs. low, HR = 0.4 [0.2, 0.9], *p* = 0.024), as well as worse FFS (high vs. low, HR = 3.5 [1.9, 6.2], *p* < 0.001), and PFS (high vs. low, HR = 4.1 [1.7, 9.9]; *p* = 0.002). However, the Sokal score did not accurately predict responses or outcomes. Male sex, lower hemoglobin concentration, and higher WBC counts were significantly associated with lower probabilities of molecular responses and/or worse outcomes (Table 2).

### Is the sokal or ELTS score a better predictor of response and outcomes?

Because of significant differences in baseline covariates between the imatinib and 2G-TKI cohorts, we used propensity-score matching to adjust subjects. In total 1332 matches were identified in the imatinib (*n* = 1064; 80%) and 2G-TKI (*n* = 268; 20%) cohorts (Table 3).

**Table 3:** Covariates in propensity score-matched cohorts.

Variable	All (*N* = 1332)	Imatinib (*n* = 1064)	2G-TKIs (*n* = 268)	*p-*value
Age, years	38 (18, 80)	38 (18, 80)	38 (18, 73)	0.664
Sex				0.878
Male	791 (59.4%)	625 (58.7%)	165 (61.7%)	
Sokal risk				0.307
Low	524 (39.2%)	429 (40.3%)	95 (35.4%)	
Intermediate	446 (33.5%)	361 (33.9%)	85 (31.7%)	
High	362 (27.3%)	274 (25.8%)	88 (32.9%)	
ELTS risk				0.153
Low	806 (60.5%)	654 (61.5%)	153 (57.2%)	
Intermediate	389 (29.2%)	311 (29.2%)	78 (29.1%)	
High	137 (10.3%)	99 (9.3%)	37 (13.7%)	
WBC, ×10E + 9/L	148 (3, 786)	145 (5, 786)	163 (6, 755)	0.287
Haemoglobin level, g/L	110 (28, 183)	111 (28, 183)	109 (57, 167)	0.289
Platelets, ×10E + 9/L	409 (36, 3707)	401 (36, 3707)	436 (80, 2887)	0.367
Blood blasts, %	1 (0, 14)	1 (0, 14)	1 (0, 13)	0.112
Blood basophil, %	5 (0, 19)	5 (0, 19)	5 (0, 19)	0.382
Ph^+^ ACA				0.892
Yes	40 (3.0%)	32 (3.0%)	8 (3.0%)	
≥ 1 Comorbidity(ies)	463 (34.8%)	375 (35.2%)	88 (32.8%)	0.620
Follow-up months	54 (3, 178)	55 (3, 178)	45 (3, 164)	<0.001

The data are presented as the number (%) or median (range), except where otherwise noted.

2G-TKI second-generation tyrosine kinase inhibitor, Ph^+^ ACA additional chromosomal aberrations in Philadelphia-positive cells, PLT platelet, TKI tyrosine kinase inhibitor, WBC white blood cell.

**Fig. 5:**
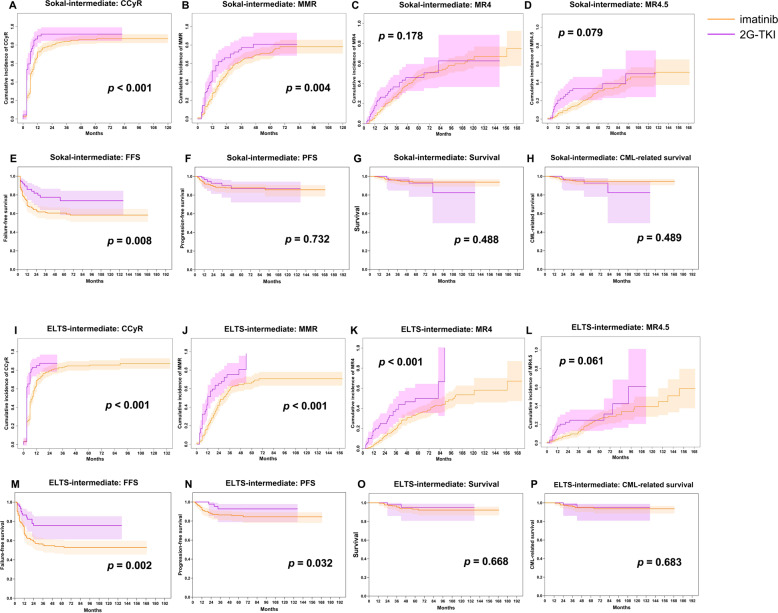
Therapy responses and outcomes in intermediate-risk subjects after the propensity-score matching. **A**–**D** CCyR, MMR, MR^4^, and MR^4.5^ by Sokal score, **E-H** FFS, PFS, CML-related survival, and survival by Sokal score, **I**–**L** CCyR, MMR, MR^4^, and MR^4.5^ by ELTS score, **M**–**P** FFS, PFS, survival, and CML-related survival by ELTS score.

Median follow-up was 55 months (IQR, 30–85 months) in the imatinib cohort and 46 months (IQR, 20–64 months; *p* < 0.001) in the 2G-TKI cohort. There were no significant differences in FFS, PFS, CML-related survival, or survival in the low- and high-risk cohorts defined by either the Sokal or ELTS scores whether subjects received initial imatinib or a 2G-TKI, except for probabilities of cytogenetic and/or molecular responses (Supplementary Figs. 1–2). However, in the intermediate-risk cohort defined by either the Sokal or ELTS scores, subjects receiving initial 2G-TKI therapy had higher probabilities of CCyR, MMR, and MR^4.5^ and a better FFS compared with those receiving initial imatinib. Better MR^4^ and PFS were detected only with the ELTS score (*p* < 0.001 and *p* = 0.032). However, initial TKI therapy had no impact on CML-related survival or survival using either the Sokal or ELTS scores (Fig. 5). We did not analyze interval to stopping TKI therapy or success rate of therapy-free remission.

## Discussion

We compared predictive accuracies of the Sokal and ELTS scores in 1661 subjects with chronic-phase CML. We found that the ELTS score was a better overall response and outcome predictor, especially in subjects receiving initial 2G-TKI therapy. Based on HRs and CIs in multivariable analyses, the ELTS score was a better discriminator between risk cohorts than the Sokal score.

Our data are consistent with some previous findings [10–16]. The study by Pfirrmann and colleagues reported that the ELTS was a better survival predictor than the Sokal score [8, 14]. However, our study focused on FFS rather than survival. As such, it is more likely to be of use to physicians in choosing the best initial TKI therapy. Geelen et al. reported that the ELTS score identified significant differences in probabilities of MMR, CML-related death, and survival in subjects receiving 2G-TKIs compared with the Sokal score [11]. We found that the ELTS score predicted probabilities of CCyR, MMR, MR^4^, FFS, and PFS in subjects receiving initial 2G-TKI therapy but not MR^4.5^. However, the ELTS score was not predictive of CML-related survival or survival. Discordances between our data and those of Geelen et al. might result from the younger age of our subjects, which is an independent predictive covariate for survival in many studies [9–16]. Also, these studies may not have been comparable for therapies given after initial 2G-TKI therapy. It is not surprising that the ELTS score is a better predictor of responses and outcomes of TKI therapy, because it was derived from a dataset of subjects receiving TKI therapy whereas the Sokal score was developed in a dataset of subjects receiving other therapies. As such, the Sokal score is best considered prognostic rather than predictive score better reflecting CML biology than therapy.

We found fewer non-CML-related deaths compared with other studies [9–16]. There are several possible explanations, including the younger age of our subjects who would be expected to be otherwise healthier, have fewer comorbidities, and therefore fewer competing causes of death [23, 24]. Also, as a tertiary referral center, there are likely subject-selection biases. For example, persons with substantial other health problems were less likely to travel to our center.

One potentially problematic area is defining failure. In our literature review, we found no consistent definition. We used definitions proposed in the 2020 ELN CML recommendations [17]. Because there was no consensus definition of accelerated phase, we analyzed our data including and excluding subjects in whom progression to accelerated phase was the failure event. Our conclusions were unchanged.

Several studies report that initial 2G-TKIs are associated with faster cytogenetic and molecular responses compared with imatinib and with lower rates of progression, especially in persons with Sokal intermediate- and high-risk scores [25–31]. However, this advantage for 2G-TKIs does not translate into better PFS or CML-related survival or survival. 2G-TKIs are recommended for initial therapy of intermediate- and high-risk cohorts in the National Comprehensive Cancer Network (NCCN) clinical practice guidelines based on the risk of progression rather than PFS, CML-related survival, or survival [18]. This differs from the ELN 2020 recommendation that does not suggest a TKI preference based on risk cohort [17]. Complicating the NCCN recommendation is the question which predictive score should be used to classify someone as intermediate- or highrisk.

In our propensity-matching analyses, in subjects classified as intermediate risk using the Sokal or ELTS scores, we found that initial 2G-TKI therapy improved that proportions of CCyR, molecular responses, and FFS compared with initial imatinib therapy but not CML-related survival or survival. In subjects classified as intermediate risk using the ELTS but not the Sokal score, initial therapy with a 2G-TKI resulted in better PFS but not better CML-related survival or survival. This finding may influence TKI-therapy decisions for physicians focused on surrogate endpoints. Why 2G-TKIs had no advantage in high-risk subjects identified by both scores could reflect relatively few subjects but also no favorable impact of 2G-TKIs when disease biology is highly unfavorable.

Consistent with several studies, we found that females had better molecular responses to TKI therapy than males and lower probabilities of therapy failure and transformation to accelerated and blast phases [32–34]. This advantage might reflect different compliance or leukemia biology or other factors [35]. Similar to previous studies, we found a lower hemoglobin concentration and higher WBC counts were associated with worse responses and/or outcomes [36–39].

Our study has limitations. First, it is retrospective. Second, we lacked a validation cohort. Third, the number of subjects receiving initial 2G-TKI therapy was only 282. Fourth, 2G-TKIs were available only after 2011 resulting in an imbalance in follow-up. Also, therapy options for subjects failing imatinib before 2011 were restricted. Fifth, use of imatinib vs. a 2G-TKI was not random nor pre specified. As such, there are likely selection biases which we tried to account for propensity-score matching. We accept this is an imperfect simulation of a randomized controlled trial. Sixth, our subjects were younger than in most other CML studies in persons of predominantly European descent and need validation in these populations. Seventh, our data are from a specialized tertiary CML center with subjects coming from all over a large country. This obviously introduces subject-selection biases. Eighth, we did not consider other 2G-TKIs approved for initial therapy, including bosutinib and radotinib. Whether our conclusions apply to these drugs is unknown. Ninth, we did not analyze interval to stopping TKI therapy or success rate of therapy-free remission. Last, we did not monitor adherence to TKI therapy which may have differed for different TKIs.

In conclusion, we found better overall prediction accuracy for the ELTS score compared with the Sokal score in persons with chronic-phase CML receiving TKI therapy, especially those receiving 2G-TKIs. People identified as intermediate risk in the ELTS score may benefit from 2G-TKI therapy compared with imatinib in achieving surrogate endpoints but not in CML-related survival or survival. The interval from start to stopping TKI-therapy and success rates of therapy-free remission were not compared.

## Supplementary information

Supplemental materials
