# Supplementary material for: Is the Sokal or EUTOS long-term survival (ELTS) score a better predictor of responses and outcomes in persons with chronic myeloid leukemia receiving tyrosine-kinase inhibitors?
Source: Leukemia. 2021 Aug 19;36(2):482–91. doi: 10.1038/s41375-021-01387-y (PMC8807391; doi:10.1038/s41375-021-01387-y)
Supplement: Supplementary file 1 — Supplemental materials [file 41375_2021_1387_MOESM1_ESM.doc]

**Supplement Figure 1. Therapy responses and outcomes in low-risk subjects after the propensity score matching.** (A, B, C and D) CCyR, MMR, MR^4^ and MR^4.5^ by Sokal score; (E, F, G and H) FFS, PFS, CML-related survival and survival by Sokal score; (I, J, K and L) CCyR, MMR, MR^4^ and MR^4.5^ by ELTS score; (M, N, O and P) FFS, PFS, survival and CML-related survival by ELTS score.





**Supplement Figure 2. Therapy responses and outcomes in high-risk subjects after the propensity score matching.** (A, B, C and D) CCyR, MMR, MR^4^ and MR^4.5^ by Sokal score; (E, F, G and H) FFS, PFS, CML-related survival and survival by Sokal score; (I, J, K and L) CCyR, MMR, MR^4^ and MR^4.5^ by ELTS score; (M, N, O and P) FFS, PFS, survival and CML-related survival by ELTS score.
